# The agreement and repeatability of measurements of ankle joint dorsiflexion and poplietal angle in healthy adolescents

**DOI:** 10.1186/s13047-022-00572-1

**Published:** 2022-09-07

**Authors:** Krzysztof Pietrzak, Izabela Miechowicz, Krzysztof Nowocień, Bartosz Kraszewski, Piotr Rzymski

**Affiliations:** 1 Department of Spondyloorthopedics and Biomechanics of the Spine, Poznan University of Medical Sciences, Poznań, Poland; 2 Department of Computer Science and Statistics, Poznan University of Medical Sciences, Poznań, Poland; 3 Department of Orthopaedics and Traumatology, Poznan University of Medical Sciences, Poznań, Poland; 4 Faculty of Health Sciences, Poznan University of Medical Sciences, Poznań, Poland; 5 Department of Environmental Medicine, Poznan University of Medical Sciences, 60-806 Poznań, Poland; 6 Integrated Science Association (ISA), Universal Scientific Education and Research Network (USERN), 60-806 Poznań, Poland

**Keywords:** Ankle joint dorsiflexion, Popliteal angle test, Reliability, Agreement, Goniometry

## Abstract

**Background:**

The intra-rater repeatability and inter-rater agreement of orthopaedics measurements are important for estimating injury risk and appropriate treatment. In clinical practice, it is often unavoidable to trust the measurements of other health professionals.

**Methods:**

This study tested the agreement and repeatability of measurements of the dorsiflexion of the foot, dorsiflexion with 90-degrees knee flexion, and popliteal angle test in healthy adolescents performed twice by three raters differing in clinical experience. Three raters, i.e., an orthopaedics specialist (16 years of experience), a resident medical doctor in orthopaedics (4 years of experience), and a physiotherapy student (1 year of experience) measured the ankle joint dorsiflexion and the popliteal angle in 142 healthy adolescent subjects.

**Results:**

The student outperformed more experienced raters by displaying good repeatability for all the evaluated parameters. The orthopaedics specialist failed to replicate the measurements of the left ankle joint passive dorsiflexion and the left popliteal angle. The medical resident in orthopaedics displayed a lack of repeatability in evaluating the right ankle joint dorsiflexion with the knee joint bent. Kendall’s W value for all parameters ranged 0.66–0.78, indicating a good inter-rater agreement.

**Conclusions:**

The study highlights that measurements of the ankle joint dorsiflexion and popliteal angle test by different health professionals can generally be trusted. It indicates that novice health professionals could potentially evaluate such parameters in healthy subjects without a quality loss.

## Background

“Practice makes better”– this common and old saying is a basic rule taught to every resident starting their training. This is certainly true in the case of various fields, including medical biology and medicine. Appropriate training, increasingly often supported by simulation-based medical education, are essential to perform qualified, accurate and standardized measurements and procedures [1–3]. On the other hand, health professionals, including experienced clinicians, are frequently overworked, forced to switch tasks, or perform concurrent multitasking. These can have varying detrimental effects on task performance and increase the risk of error [4–7]. Moreover, experienced health professionals may more frequently [8–10] be subject to some biases, among which the most common include anchoring bias (the tendency to rely on the pre-existing assumptions when making clinical decisions), availability bias (the tendency to weigh the likelihood of things by how easily they are recalled), and confirmation bias (the tendency to give greater weight to data that support a preliminary diagnosis while failing to seek or dismissing contradictory evidence).

Having some procedures, including basic screening and diagnostic tests, performed by more novice health professionals, or under some circumstances, even medical students may decrease the work overload for experienced healthcare workers [11–13]. This, however, requires first to ensure that such examinations can be performed at the appropriate level of accuracy and repeatability.

In orthopaedics, accurate and repeatable measurements for ankle joint dorsiflexion and popliteal angle can be used to estimate injury risk and plan appropriate treatment in case of discrepancies [14, 15]. The shortening of the posterior thigh muscles, which is one of the elements influencing the size of the popliteal angle, increases the risk of knee injuries, and especially in adolescents, can lead to back pain, as well as asymmetry in the structure of the back [16–18]. The ankle joint dorsiflexion and knee range of motion may change during growth [16]. Additionally, the muscular fascicle length and the tendon stiffens, which impact the ankle and knee range, may change during growth, and according to some authors, may also be influenced by stretching [17–19]. Nevertheless, their measurement would be valuable to determine if ankle joint dorsiflexion and popliteal angle test changes have occurred within an individual over time and due to exercise [17, 18]. However, the accuracy and repeatability of the range of motion can vary depending on the method and potentially on the clinician’s experience [20]. Previous studies have suggested that being a more novice health professionals may not always be an obstacle to performing some medical procedures [21]. It is important to emphasize that the ankle joint dorsiflexion and popliteal angle tests are considered as the most valuable methods in goniometric measurements [22, 23].

The present study aimed to test the agreement and repeatability of measurements of the ankle joint dorsiflexion and popliteal angle test in adolescents performed twice by three raters differing in clinical experience: an orthopaedics specialist (male, 16 years of experience in goniometry and patients examination, 41 years old), a resident medical doctor in orthopaedics (male, 4 years of experience in goniometry and patients examination, 29 years old), and a physiotherapy student (male, 1 year of experience in goniometry and patients examination, 24 years old). We hypothesized that the more experienced the rater, the better repeatability of the measurement.

## Methods

### Subjects

The study group consisted of 142 (57 female, 85 male) adolescents attending the junior high school in Poznań, Poland (age 13–15, mean ± SD 13.8 ± 1.0). The inclusion criteria were as follows: no orthopaedics and/or neurological condition, practicing sports only at school, attending standard curriculum (SC) or extended physical activity curriculum (EPAC). Only healthy participants were included in the study because many orthopaedics conditions, and particularly neurological and neuro- orthopaedics disorders, are accompanied by spasticity, a phenomenon that reduces the range of joint motion and deforms the lower limb [24–26].

Overall, 60 and 82 subjects attending EPAC and SC were recruited, respectively. At the time of the study, there was a total of 5117 junior high school attendees in Poznań (although a share of healthy subjects was not possible to estimate). This considered the representativeness of the sample size was calculated with Cochran’s formula [27]. A power calculation indicated that for the considered sample size (*n* = 142) a margin error was 8.1% at the confidence level of 95%.

The EPAC subjects had 14–18 physical education classes per week, while SC had four classes, 45 min each, starting with a few-minute warm-up including running, squats, and static stretching. The sports practiced during the classes included football, basketball and field hockey. The study protocol was approved by the Bioethical Committee of the Poznan University of Medical Sciences (Approval No. 212/17). All parents and school heads gave their written consent for the study. The subject provided verbal consent before the examinations. Three subjects did not agree to participate in the study, despite the written consent of their parents – all were excluded from the examination. All were advised on the purpose and course of the investigations and were given free will to withdraw from the examination at any time.

### Measurements

The ankle joint dorsiflexion and ankle joint dorsiflexion with a knee in 90 degrees of flexion and the popliteal angle test were measured in all subjects twice within 2 h interval. A need for such a short interval in studies of repeatability was acknowledged in previous research [28, 29]. Each rater performed twice testing up to no more than 15 students a day to avoid fatigue factor. Examination of 142 individuals was performed during 11 visits to schools. Three individuals were asked to enter the examination room at each examination. Afterward, they were informed to return to classes and return to the examination room after 2 h. Each time, the measurements were taken by three rater who underwent 2 weeks of training of patient examinations, conducted in the orthopaedics ward.

The results recorded by one rater were not available to others during the study. Similarly, the results recorded during the first test were unavailable for a rater before a second measurement series. Before the examinations, the raters were double pre-checked in terms of quality and skill of the testing by the independent specialists in orthopaedics who did not participate in the study. According to the provided opinion, all three raters were performing the examination correctly.

All examined subjects did not have physical activity classes during the day of testing. All examinations were performed with students lying on a mattress. The maximum passive range of ankle joint dorsiflexion was checked in a supine position with lower extremities extended. Ankle joint dorsiflexion was evaluated with the hip and knee joints flexed to 90 degrees. During the examination, it was ensured that the dorsiflexion was in the ankle to eliminate the action of the middle and forefoot. The raters took special care to perform the dorsiflexion in the neutral position of the ankle and foot, without any inversion or eversion. The last test was a popliteal angle test (maximum extension of the knee joint with the hip flexed to 90 degrees).

**Fig. 1:**
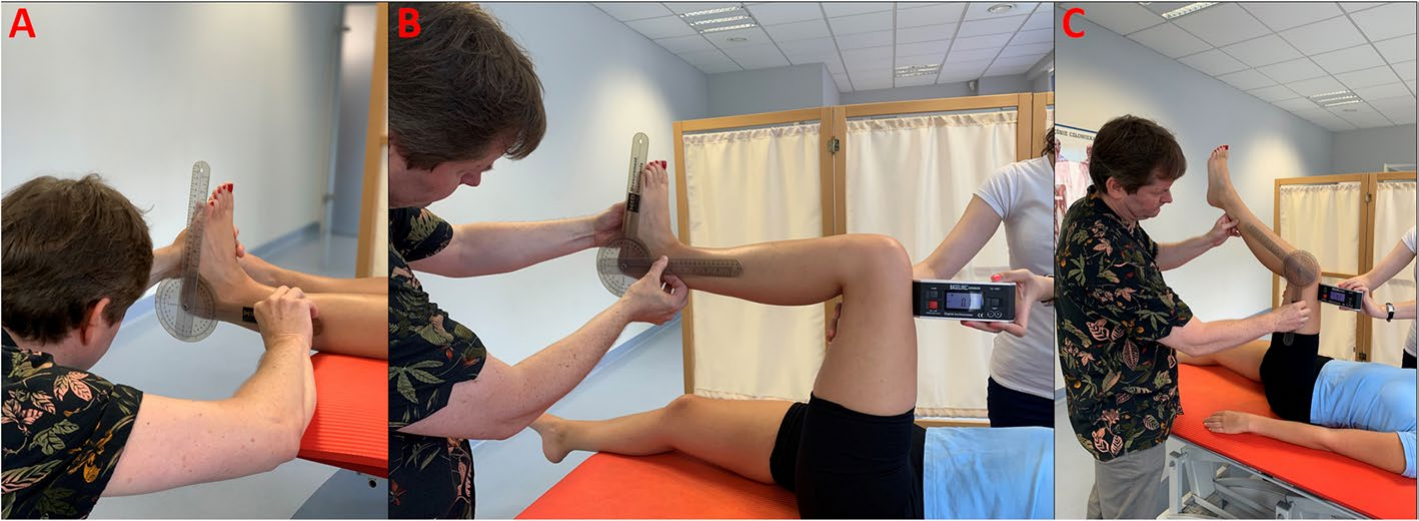
The schematic presentation of **A** ankle joint dorsiflexion of the foot measurement, **B** ankle joint dorsiflexion of the foot with 90-degeses of knee flexion measurement, and **C** Popliteal test angle measurement

The ankle joint dorsiflexion was measured using landmarks: the proximal (the fibular shaft and over the lateral malleolus) and the distal (the shaft of the fifth metatarsal). The axis of the goniometer was distal to, but in line with, lateral malleolus at the intersection of lines through the lateral midline of the fibula and the lateral midline of the fifth metatarsal. The same landmarks and goniometer axis were applied for the measurement of ankle joint dorsiflexion with 90 degrees-knee flexion. The assistant stabilized (pre-instructed school nurse) the knee in the position found by the rater before. The assistant also was holding the electronic inclinometer (baseline digital inclinometer) to observe the 90 degrees of flexion of the hip. However, it was a rater observing. To measure the popliteal angle: The hip flexion was flexed 90 degrees, additionally confirmed with an electronic inclinometer. The goniometer was held alongside the thigh, pointing to the great trochanter, with the second landmark alongside shin to the lateral malleolus. The goniometer axis was the lateral condyle of the femur. Additionally, the assistant stabilized the knee in the position found by the rater, and was holding the inclinometer. These measurement techniques are considered the most reliable [30–32]. Their scheme is presented in Fig. 1.

During all examinations, the hip and knee were stabilized by the pre-instructed nurse. The subjects did not wear socks and wore loose shorts, which did not restrict movement. Both lower limbs were examined. The range of motion was measured with a universal goniometer (Merck, Darmstadt, Germany) because it is a widely used and accepted instrument in orthopedics, while previous studies have shown that the use of this method in the clinical evaluation of ankle joint dorsiflexion, knee examination, especially active knee extension test, and popliteal angle is reliable [22, 23, 33]. The range of the popliteal angle and ankle joint dorsiflexion was expressed in degrees.

### Statistical analyses

The statistical analysis was performed using Statistica 12 (StatSoft Inc., Tulsa, OK, USA) and PQStat (PQStat Software, Poznań, Poland) and *p* < 0.05 was considered as statistically significant. The assumption of the Gaussian distribution was evaluated with the Shapiro-Wilk test. To assess the reliability of rater scores, the stability of scores and the agreement were analyzed. To evaluate the stability, the test-retest reliability method was used: the same group was tested twice using the same measurement tool, and stability was shown by high repeatability. To that end, we analyzed each rater’s scores for changes between examination I and II using the Wilcoxon test because the analyzed variables were not normally distributed. The Spearman correlation coefficients (Rs) were also calculated. Kendall’s coefficient (W) was calculated for the first and the second examination to determine the agreement between the scores from the three raters (a specialist in orthopedics, a resident medical doctor in orthopedics and a physiotherapy student), Kendall’s W lower than 0.4 was considered as insufficient agreement, Kendall’s W in the range (0.40; 0.60) was rated as satisfactory agreement; (0.60; 0.80) as good agreement, and Kendall’s W above 0.80 was considered to be a very good agreement [34]. The analysis was performed for each evaluated aspect.

## Results

### Repeatability

The summary of measurement results obtained by each rater is provided in Table 1. The results of the repeatability of scores obtained by three raters are summarized in Table 2. In the case of orthopedic specialists, the lack of repeatability of scores was found for passive ankle joint dorsiflexion of the left foot and the left popliteal angle. For other parameters, the repeatability was retained. The medical resident in orthopedics displayed a lack of repeatability for evaluation of the right ankle joint dorsiflexion with the knee joint bent. Repeatability was demonstrated for all the other aspects. The physiotherapy student showed the best performance with the repeatability found for all the evaluated parameters.

**Table 1:** The results (mean ± SD) of measurements of the ankle joint dorsiflexion, ankle joint dorsiflexion with 90-degrese knee flexion and the popliteal angle (°) performed by each rater in 142 healthy adolescent subjects examined twice within 2 h

Parameter	Examination	Orthopaedics specialist	Resident medical doctor in orthopaedics	Physiotherapy student
Right ankle dorsiflexion	First	7.8 ± 5.1 (0–20)	9.7 ± 4.7 (0–24)	8.5 ± 4.4 (0–20)
	Second	8.0 ± 5.5 (0–22)	9.5 ± 4.5 (0–24)	8.9 ± 4.1 (0–20)
Left ankle dorsiflexion	First	7.18 ± 5.42 (0–25)	9.95 ± 4.86 (0–26)	8.7 ± 4.0 (0–20)
	Second	8.0 ± 5.0 (0–21)	10.0 ± 4.7 (0–24)	9.2 ± 4.1 (0–20)
Right ankle dorsiflexion with knee flexion	First	17.7 ± 6.1 (4–35)	18.1 ± 5.8 (4–32)	18.0 ± 5.3 (4–32)
	Second	17.0 ± 5.7 (7–32)	17.3 ± 5.9 (4–36)	17.6 ± 5.6 (4–37)
Left dorsiflexion, with knee flexion	First	15.7 ± 6.7 (0–40)	16.7 ± 6.4 (4–32)	17.2 ± 5.4 (4–32)
	Second	16.1 ± 5.8 (4–32)	16.4 ± 6.2 (4–38)	17.3 ± 5.8 (0–32)
Right popliteal	First	33.9 ± 15.5 (0–70)	29.2 ± 12.0 (4–60)	29.5 ± 10.6 (6–58)
	Second	34.0 ± 14.4 (0–70)	29.0 ± 11.3 (4–68)	29.6 ± 10.1 (6–52)
Left popliteal	First	37.9 ± 13.7 (0–70)	30.5 ± 11.5 (5–60)	30.4 ± 9.6 (2–54)
	Second	36.0 ± 14.0 (0–66)	30.0 ± 11.3 (6–52)	31.4 ± 11.0 (4–58)

**Table 2:** Repeatability of scores of ankle joint dorsiflexion, ankle joint dorsiflexion with 90-degreses knee flexion and polietal angle, obtained by three raters with varying levels of clinical experience in 142 healthy adolescent subjects examined twice within 2 h

Parameter	Orthopaedics specialist	Resident medical doctor in orthopaedics	Physiotherapy student	Orthopaedics specialist	Resident medical doctor in orthopaedics	Physiotherapy student
	Wilcoxon Test ***p***-value			Spearmans Rs coefficient (***p***-value)		
Right ankle dorsiflexion	0.823	0.347	0.108	0.710 (< 0.001)	0.663 (< 0.001)	0.715 (< 0.001)
Left ankle dorsiflexion	0.021	0.936	0.068	0.702 (< 0.001)	0.722 (< 0.001)	0.727 (< 0.001)
Right ankle dorsiflexion with knee flexion	0.803	0.040	0.143	0.428 (< 0.001)	0.680 (< 0.001)	0.761 (< 0.001)
Left ankle dorsiflexion with knee flexion	0.512	0.643	0.972	0.464 (< 0.001)	0.791 (< 0.001)	0.748 (< 0.001)
Right Popliteal	0.789	0.946	0.932	0.675 (< 0.001)	0.686 (< 0.001)	0.747 (< 0.001)
Left Popliteal	0.032	0.436	0.700	0.609 (< 0.001)	0.716 (< 0.001)	0.742 (< 0.001)

### Agreement

The Kendall’s W values ranged from 0.63 to 0.78 depending on parameter, indicating good intrarater agreement (Table 3).

**Table 3:** Power of raters’ agreement of scores of measurement in first and second examination (*n* = 142) evaluated using Kendall’s W

Parameter	Examination	Kendall’s W
Right ankle dorsiflexion	First	0.69
	Second	0.69
Left Right ankle dorsiflexion	First	0.71
	Second	0.66
Right ankle dorsiflexion with knee flexion	First	0.66
	Second	0.63
Left ankle dorsiflexion with knee flexion	First	0.66
	Second	0.67
Right popliteal	First	0.78
	Second	0.78
Left popliteal	First	0.73
	Second	0.75

## Discussion

The present study provides insight into the repeatability and agreement of the measurements of the ankle joint dorsiflexion and popliteal angle test provided by three raters who differed in clinical experience. As revealed, the measurements undertaken by an experienced specialist were not repeatable for two out of all six parameters of examination, both for the left side. It may potentially be a chance finding. All the raters were right-handed, while the examination was performed in a similar fashion on both sides. A resident in orthopaedics displayed the lack of repeatability of one of parameter (evaluation of the right ankle joint dorsiflexion),, while the physical student outperformed the rest of the raters not only in repeatability but also reliability of measurements as demonstrated by the highest values of Spearman’s correlation coefficient. Previous studies are evidencing that clinical experience is not, in selected situations, related to better performance [20, 35]. For example, Borstad and Briggs have shown no difference between novice and experienced clinicians in a latissimus dorsi length measurement [20]. Morgan and Cleave-Hogg indicated that clinical experience had no predictive value in performance assessments when using standardized anesthesia simulation scenarios [35]. The observation of the present study may have different explanations. It may arise from the assumption of orthopaedics specialists that slight differences in angle measurements will have little significance for a further clinical course, in particular for future treatment. In turn, a student’s may perform best due to his potential belief in the need for a thorough examination to yield accurate and clinically relevant results. By no means the present paper intends to challenge the significance of clinical experience in the accuracy and repeatability of medical measurements. Numerous works are showing that more advanced techniques require training, particular skills and knowledge [36–38]. Although since the evaluation of parameters considered in our study is not highly challenging, it is worth highlighting that novice individuals could assess them without a quality loss.

The second objective of the present study was to evaluate the level of agreement between measurements performed by different rater during both examination series. This is important in clinical research and practice as it is frequently needed to trust in results provided by other health professionals. Although some previous studies on the inter-rater agreement in orthopaedics measurements indicated a very good or even excellent level as high as 95%, it should be noted that it is likely a result of a small number of tested samples/individuals, e.g., 15 radiographs [39], 7 cadaver specimens [40] or 20–25 patients [41, 42]. At the same time, it was highlighted that such analyses are only valid if the number of subjects is at least 50 [43]. The reliability of measurements of goniometry of knee and foot range of motion reported in some of the previous studies is higher compared to that in our research [22, 23, 33, 44]. However, all these studies investigated reliability on small sample size. Research involving a greater number of subjects had similar reliability to that obtained in our study [33].

A negative aspect of the test-retest reliability method is the interval between the test and re-test. When the interval is too short, the rater may remember the scores and give similar scores on the re-test, which will increase the value of the correlation coefficient. If the interval between the measurements is too long, the correlation coefficient may be lower. In our study, correlation coefficients for all the raters and most aspects suggested a significant relationship (R_S_ = 0.6–0.8). This justifies the claim that the interval between the first and second examination was adequate. Importantly, the second Kendall’s W value was slightly lower for three aspects and slightly higher for three aspects in comparison to the first examination. This suggests that despite the 2 h passed between the examinations, the raters scored patients independently of one another, without any consultations after the first examination.

An increase in the number of subjects can result in a decrease in agreement level. For example, in one study encompassing 60 healthy subjects, the inter-rater agreement in measurements of small angles of dorsiflexion was classified only as fair ([45]. The present study examined a total of 142 patients, three-fold the threshold recommended for measurements of reliability [43]. Despite the differences in repeatability of selected parameters related to the rater’s medical experience, shown in the previous sub-section, the agreement results indicate that measurements of the ankle joint dorsiflexion and popliteal angle test performed by different physicians can generally be trusted.

Study limitations must be considered. The research included only healthy individuals. This is because various orthopedic disorders are accompanied by the spasticity phenomenon, which can dynamically change within a short period of time and could even be influenced by the sole examination. Therefore, including subjects with pathologies could bias the findings, their interpretation and conclusions. However, it cannot be entirely ruled out that examination of healthy subjects also influenced the results, e.g., through an assumption of orthopedic specialist that slight differences in angle measurements will have little significance for a further clinical course. It remains unknown whether novice health professionals could evaluate similar parameters in disabled subjects without quality loss - this would require an additional, specifically designed study.

## Conclusions

The present study showed that more novice physicians could potentially perform selected orthopaedics examinations of healthy subjects without a quality loss. Further studies employing a larger number of compared raters and disabled patients are required to confirm this conclusion. As demonstrated, the precision of the evaluation had a significant impact on the score, while the effect of the rater’s professional experience was smaller. A least experienced rater, a student of physical therapy, revealed the highest repeatability of measured parameters.

## Data Availability

The datasets used and analysed during the current study are available from the corresponding author on reasonable request.
